# Dr. Rufus A. Egbert

**Published:** 1918-12

**Authors:** 


**Dr. Rufus A. Egbert,** died of influenza Oct. 29 at Custer City, Pa.. where he had practiced for 40 years. He was 69 years old.

